# Heat shock proteins in farmed aquatic animals: advancing animal health and welfare management through building resilience

**DOI:** 10.3389/fimmu.2026.1795910

**Published:** 2026-08-03

**Authors:** PPSK Patabandi, Parisa Norouzitallab, Dipesh Debnath, Rishikesh Subhashrao Dalvi, Amit Kumar Sinha, Mazlan bin Abd Ghaffar, Yeong Yik Sung, Kartik Baruah

**Affiliations:** 1 Higher Institution Centre of Excellence (HICoE), Institute of Climate Adaptation and Marine Biotechnology (ICAMB), Universiti Malaysia Terengganu, Kuala Nerus, Terengganu, Malaysia; 2 Department of Livestock Production, Faculty of Agricultural Sciences, Sabaragamuwa University of Sri Lanka, Belihuloya, Sri Lanka; 3 Department of Animal Nutrition and Management, Faculty of Veterinary Medicine and Animal Sciences, Swedish University of Agricultural Sciences, Uppsala, Sweden; 4 ICAR-Central Inland Fisheries Research Institute, Regional Centre, Guwahati, India; 5 Department of Zoology, Maharshi Dayanand College of Arts, Science and Commerce, Mumbai, India; 6 Department of Aquaculture and Fisheries, University of Arkansas at Pine Bluff, Pine Bluff, AR, United States

**Keywords:** diseases, heat shock protein, infection stress, robustness, sustainable aquaculture

## Abstract

Aquaculture is vital for global food security. Intensification of farming practices has compromised the welfare of farmed aquatic animals and increased disease outbreaks. Conventional treatments, such as antibiotics, face growing challenges due to antimicrobial resistance and environmental concerns. Consequently, there is a pressing need for sustainable, welfare-oriented disease management strategies in aquaculture animals. One promising solution involves heat shock proteins belonging to the 70-kDa family [Hsp70s], which provide several health benefits. Hsp70s are evolutionarily conserved proteins produced constitutively or induced in response to various conditions, where they function as molecular chaperones to perform immune-enhancing functions vital for cellular survival during both stable and stressful conditions. Recent advances have highlighted the role of Hsp70s and their inducers in promoting disease tolerance by building resilience and improving overall animal welfare in aquaculture systems. This review integrates current knowledge on the role of Hsp70s in fish stress physiology and welfare and critically evaluates their potential applications as sustainable tools for improving fish health, enhancing welfare outcomes, and controlling disease outbreaks in aquaculture systems.

## 1 Introduction

Aquatic finfish and shellfish are fundamental to global food and nutritional security, contributing approximately 20% of animal protein consumed worldwide, as well as essential fatty acids and micronutrients vital for human health (1). The production of aquatic foods from the captured fisheries sector has remained largely stagnant for more than a decade due to overexploitation, habitat degradation, and climate-related stressors (2, 3). On the contrary, the aquaculture sector has expanded rapidly, with production increasing from 34 Mt in 1997 to 130 Mt in 2022 (4). This growth has improved the year-round availability of a wide variety of nutritionally rich aquatic foods among consumers from low-, middle-, and high-income countries (5–7). Despite this remarkable expansion, the sector faces mounting environmental and biological challenges that threaten its long-term sustainability and capacity to meet growing global demand (8).

**Figure 1:**
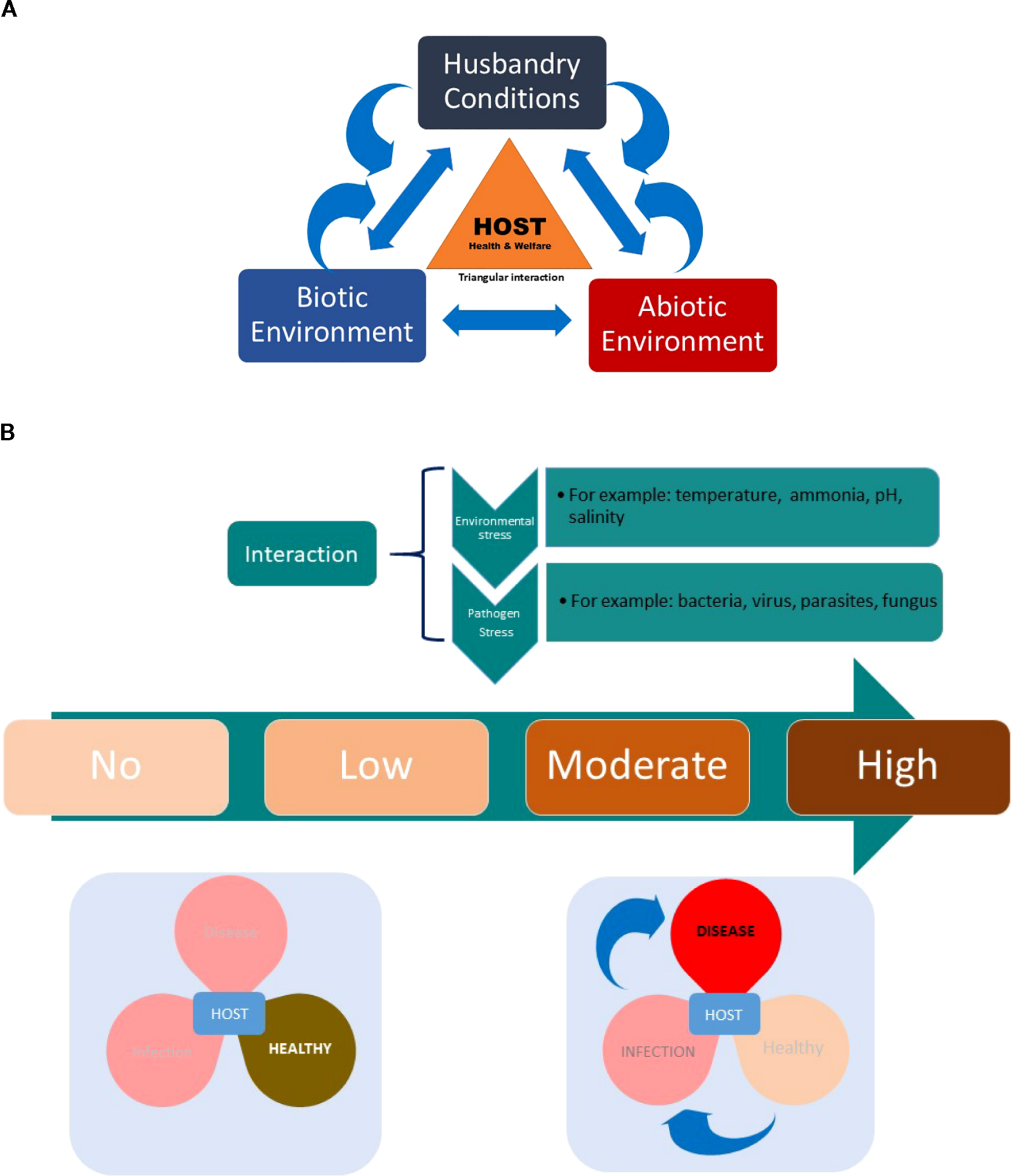
**(A)** Conceptual framework illustrating the triangular interaction among the host, biotic environment and abiotic environment that collectively regulate host health condition and welfare in aquaculture animals. Disease outcomes are influenced by host factors (e.g., immune status and physiological condition), biotic factors (e.g., pathogens, parasites, and microbiota), abiotic factors (e.g., temperature, water quality, and dissolved oxygen), and husbandry practices (e.g., stocking density, feeding, and hygiene). **(B)** A schematic representation of the combined effects of environmental and pathogen stresses on aquaculture animals. Simultaneous exposure to these stressors can compromise the defence response of the cultured organisms and increase susceptibility to infection and disease outbreaks, depending on the intensity and duration of the combined stresses.

Among these challenges, climate change and farming intensification are reshaping the production landscape of the sector. Rising water temperatures, increased frequency of heatwaves, hypoxia, and altered salinity regimes are increasingly exposing farmed aquatic animals to chronic and acute physiological regimes (9–12). These environmental stressors disrupt physiological homeostasis, impair growth and metabolism, compromise welfare and suppress immune function, thereby reducing the capacity of farmed species to withstand pathogenic challenges. An example of such cases is acute hepatopancreatic necrosis disease (AHPND) in penaeid shrimp, where elevated temperatures and poor water quality were found to enhance the virulence of the causative bacterium, *Vibrio parahaemolyticus*, while simultaneously weakening host defences, resulting in severe disease outbreaks and high mortality (13–15). Similarly, elevated water temperatures have been associated with increased replication and disease severity of infectious salmon anaemia virus (ISAV) in Atlantic salmon. These observations reinforce the fact that disease emergence in aquaculture arises from a complex interplay among the host, biological agents/pathogens, and environmental factors rather than from pathogens alone (**Figure 1**, 16–18). Environmental perturbations simultaneously weaken host defences and change the structure of microbial communities, shifting normally balanced host–microbe interactions toward opportunistic or pathogenic states that increase disease susceptibility and compromise production performance (19, 20). Consequently, environmental stress often acts as a critical determinant of disease susceptibility, outbreak severity and production losses in aquaculture systems.

Historically, disease management in aquaculture has relied on the use of antibiotics. However, their widespread and often misuse has accelerated the emergence of antimicrobial resistance, reducing subsequent treatment efficacy (21–23). This misuse also poses serious risks to food safety, human health and the environment through the spread of resistance genes and antibiotic residues (9, 24–26). As aquaculture products are traded globally, these risks extend beyond producing countries and represent a significant transboundary public health concern (27, 28). Moreover, antibiotics offer little protection against environmental stressors and are ineffective against viral infections and parasitic diseases, highlighting the need for broader health management approaches that enhance host robustness rather than targeting individual pathogens. This need is particularly important for aquaculture species, where protection against disease relies predominantly on innate immune mechanisms. Unlike mammals, shrimp lack adaptive immunity altogether, while fish depend predominantly on innate immune responses as their first and most immediate line of defence, despite possessing an adaptive immune system. Consequently, strengthening innate immunity offers a promising strategy for improving resistance against a wide range of pathogens and environmental stressors.

Given the scale and complexity of the challenges, there is a clear need for a paradigm shift in the strategies for controlling diseases in aquaculture animals. In fact, long-term sustainability requires a preventive and welfare-oriented health management framework that focuses on building the intrinsic capacity of aquatic animals to cope with environmental and biological stress. Rather than focusing exclusively on pathogen-specific interventions, future health management strategies should aim to strengthen conserved host defence pathways that protect multiple stress contexts. Such an approach is especially relevant in aquaculture, where environmental stress and infectious disease are closely linked, and innate immunity is the primary determinant of health and survival (29).

In this context, one approach is the discovery of heat shock proteins belonging to the 70-kDa family (Hsp70s), which have emerged as pivotal molecular regulators of cellular protection and stress tolerance. In this review, we synthesise current knowledge on the biological functions of Hsps, with particular focus on Hsp70s, and critically evaluate the evidence supporting their roles in immune competence, stress tolerance and disease resistance. We further examine emerging strategies for inducing Hsp70 within aquaculture species and discuss how Hsp70-mediated enhancement of host robustness could form the basis of a broad-spectrum health management framework capable of mitigating the combined impacts of environmental stressors and diverse pathogen groups, thereby advancing animal health, welfare and the sustainability of aquaculture production systems under environmental change and farming intensification.

## 2 Heat shock proteins – classification and functional significance

Heat shock proteins [Hsps], commonly referred to as stress proteins, are a group of evolutionarily conserved proteins present across all domains of life, from prokaryotes to complex eukaryotes (30–32). These proteins vary in molecular weight, typically between 16 and 110 kDa, and play essential roles in maintaining cellular integrity under both normal and adverse conditions. The discovery of Hsps originated from early observations by Ritossa (33), who demonstrated that exposure to elevated temperatures triggered distinct transcriptional activity in specific chromosomal regions of *Drosophila melanogaster*. These regions, later termed heat shock loci, were subsequently shown to encode a unique set of proteins that are rapidly induced in response to stress, which demonstrated that exposure to elevated temperatures triggered distinct transcriptional activity in specific chromosomal regions of *Drosophila melanogaster*. These regions, later termed heat shock loci, were subsequently shown to encode a unique set of proteins that are rapidly induced in response to stress (34). Although initially associated with thermal stress, it is now well established that the heat shock response represents a universal and evolutionarily conserved cellular defence mechanism activated across taxa (30). In addition to heat stress, a wide range of environmental and biological stressors, such as anoxia, bio-contaminants, ischemia, toxins, hormones, crowding, starvation, hypoxia, acidosis, infectious agents, i.e., bacteria, viruses, and fungi, could also lead to the up-regulation of Hsps in organisms (30, 35, 36). In aquaculture systems, where animals are frequently exposed to fluctuating environmental conditions and husbandry-related stressors, the heat shock response plays a central role in maintaining physiological stability and supporting stress resilience.

Importantly, Hsps are not exclusively stress induced. Many members are expressed constitutively in the normal unstressed cells and are commonly referred to as heat shock cognates [Hscs]. These constitutive chaperones represent approximately 5-10% of the total cellular protein in healthy growing cells (37). Under normal physiological conditions, the constitutive form performs important housekeeping functions associated with both protein biogenesis and protein homeostasis in the cells, including protein folding and trafficking, supporting and repairing damaged cytoskeleton elements, assisting in the production and folding of intracellular proteins, enzymes, and hormone receptors, and maintenance of mitochondria and nuclear integrity, as well as cell wall lipoprotein membranes (32, 38, 39). When cells experience stress caused by environmental and/or pathogenic biological factors, the inducible form of Hsps is rapidly upregulated, often reaching concentrations two- to three-fold higher than their constitutive counterpart, thereby enhancing the cellular capacity to cope with stress-induced damage (40, 41). Eukaryotic Hsps are classified into several families based on their function, sequence homology, and molecular mass measured in kilodaltons [kDa]. These families include 100 kDa, 90 kDa, 70 kDa, 60 kDa, and the 16–30 kDa group, and are usually referred to as Hsp100, Hsp90, Hsp70, Hsp60, and the low molecular weight class of proteins [sHsps], respectively (42–44). Most of these Hsp families include both constitutive and inducible members, with the notable exception of the low molecular weight, which are predominantly stress-inducible and do not have a counterpart constitutive chaperone within the non-stressed cells (45, 46).

Inducible Hsps play multifaceted roles during and following exposure to stress, particularly under conditions that disrupt cellular redox balance. Environmental and pathogenic stressors can lead to oxidative damage, protein misfolding, aggregation, and DNA instability, ultimately impairing cellular function. Hsps counteract these effects through several complementary mechanisms. Some Hsps, particularly those in the Hsp70 family, play a crucial role in protein sorting and quality control. They select and direct abnormal proteins to the proteasome or lysosomes for degradation, thus helping to clear damaged proteins more effectively (47–49). In some cases, where misfolded proteins need to be rescued, the same machinery modulates the cell survival pathways by interacting with components of the apoptotic machinery, thereby limiting stress-induced cell death and preserving tissue integrity (50–52). In addition, several Hsps exert immunomodulatory functions that enhance innate and adaptive immune responses, a feature of particular relevance for health and welfare management of farmed animals.

Among the different Hsp families, Hsp70 represents the most extensively studied and highly conserved group of the stress protein families (53, 54). To date, more than 120 Hsp70-related proteins have been characterised, displaying a high degree of sequence conservation across diverse taxa, including mammals, fish, and molluscs (55). Despite this significant conservation, the Hsp70 family shows substantial diversification among aquatic organisms. Multiple inducible and constitutively expressed Hsp70 paralogs have been identified in teleost fishes, crustaceans, and molluscs, reflecting lineage-specific evolutionary adaptations to diverse environmental conditions (56–59). In teleosts, this diversification has been facilitated in part by successive whole-genome duplication events, which generated additional copies of numerous stress-response genes, including members of the Hsp70 family (60–62). Following duplication, many Hsp70 paralogs underwent subfunctionalisation and neofunctionalisation, leading to divergence in their expression patterns, tissue specificity, and responsiveness to environmental stressors (61–63).

Comparative genomic and transcriptomic studies further reveal considerable interspecific variation in both Hsp70 gene repertoire and expression profiles among aquaculture species (58, 63–66). For example, several teleost species possess multiple inducible *hsp70* genes alongside constitutively expressed *hsc70* homologues, whereas crustaceans generally harbour a more limited Hsp70 repertoire but often exhibit robust inducible responses to environmental and pathogenic stressors (57, 64, 66). Such differences are likely to influence the capacity of different species to mount effective cellular stress responses and may partly explain observed variation in stress tolerance, immune responsiveness, and disease susceptibility among farmed aquatic animals.

Baseline expression levels of Hsp70 also vary substantially among species, populations, and tissues, reflecting adaptation to distinct ecological niches and environmental histories (64, 67–69). Species inhabiting highly variable or stressful environments frequently exhibit elevated constitutive expression of Hsp70 and other molecular chaperones, thereby maintaining a state of enhanced preparedness for subsequent exposure to stress (67, 68). Conversely, species adapted to relatively stable environments often rely more heavily on inducible Hsp70 responses when confronted with environmental perturbations (68–70). These differences in constitutive and inducible expression patterns are particularly relevant when evaluating Hsp-inducing interventions, as they may influence both the magnitude of Hsp70 induction and the resulting protective effects.

Variation in Hsp70 gene copy number has also been reported among aquatic taxa, although the functional implications of this variation remain incompletely understood (58, 61, 71). Nevertheless, an expanded Hsp70 repertoire may provide greater regulatory flexibility during exposure to environmental and pathogenic challenges by allowing specialised paralogs to respond to distinct stress conditions (61, 62, 70). Such genomic and functional diversity suggests that species-specific Hsp70 biology should be considered when developing robustness-based health management strategies and when interpreting the outcomes of Hsp-induction studies across aquaculture species.

The high level of conservation, together with its evolutionary diversification, highlights the biological significance of Hsp70 in managing cellular stress responses and preserving physiological balance. In aquatic productive systems, increasing evidence suggests that Hsp70 is a key determinant of stress tolerance, immune competence, and cellular recovery following exposure to environmental and pathogenic challenges. Beyond its mechanistic roles, Hsp70 is increasingly recognised as a molecular link between stress physiology and health and welfare management, as its modulation is associated with improved robustness under suboptimal farming conditions. Importantly, the predominance of innate immune mechanisms in fish and particularly in crustaceans further underscores the significance of Hsp70. Unlike mammals, which rely heavily on adaptive immunity, aquatic animals depend largely on innate defence pathways for protection against pathogens (72–74). Consequently, Hsp70-mediated enhancement of cellular protection and innate immune function represents a promising strategy for improving broad-spectrum tolerance against both environmental stressors and diverse pathogen groups. Accordingly, this review places particular focus on the Hsp70 family as a promising molecular target for the development of novel, sustainable and welfare-oriented strategies aimed at improving health and welfare management, as well as for controlling diseases in aquaculture animals. Furthermore, the biological significance of Hsp70 is discussed in relation to nutraceuticals derived from natural sources reported to induce or co-induce its production, highlighting their potential application as practical tools for building resilience and supporting proactive health and welfare management in farmed aquatic animals.

## 3 Regulation of Hsp70 expression: molecular bases and implications for health and welfare management

The expression of Hsp is strongly regulated at the transcriptional level through the activation of heat shock transcription factors [HSFs] that bind to specific promoter regions called heat shock elements [HSEs] located upstream of heat shock genes (75). A defining feature of major heat shock genes is the absence of introns, a structural characteristic that allows the rapid translation of mRNA into new proteins after exposure to stress (76). This rapid inducibility is particularly relevant in farmed aquatic animals, where sudden environmental fluctuations, such as temperature change, hypoxia, or pathogen exposure, can compromise animal health and welfare if the host stress responses are delayed or insufficient.

In vertebrates, including fish, several Hsp isoforms have been identified, although their number varies across taxa. While humans were reported to possess six HSFs [HSF1, HSF2, HSF4, HSF5, HSFX, and HSFY (75, 77), finfish, along with mice and chickens, generally express four HSFs. Among these, HSF1, HSF2, and HSF4 are ubiquitously expressed across tissues (78–80), whereas HSF3 appears to be avian-specific (81). HSF1 is widely recognised as the primary regulator of heat shock gene expression under stress episodes (40, 82, 83), while HSF2 is more selective and plays more specialised roles during early development and differentiation (82, 84). Notably, exposure to stress does not substantially change the total HSF1 protein levels; rather, it induces a functional transition of HSF1 from an inactive to a transcriptionally active state, thereby initiating the expression of Hsp70 (85, 86). In the absence of stress, HSF1 resides in the cytoplasm as an inactive monomer bound to an Hsp90 heterocomplex, which prevents DNA binding (87). When stress occurs, HSF1 is released from this complex and undergoes hyperphosphorylation through ras-dependent mitogen-activated protein kinase (MAPK) signalling pathways. This modification promotes trimerization of HSF1 into its transcriptionally active form, which translocates to the nucleus and binds to HSEs within heat shock gene promoters (87–89). Upon binding of HSF1 to its target, Hsp70 is expressed through normal transcription and translation processes, which contribute to enhancing cellular capacity to maintain protein homeostasis, mitigate damage, and support survival. However, an increased intracellular level of free Hsp70 also leads to a negative feedback loop that attenuates further HSF1 activity, thereby preventing excessive stress responses.

In this regulatory process, Hsp90 causes the inhibition of HSF1 oligomerization and DNA binding, while Hsp70 suppresses HSF1, ensuring balanced stress signalling (75). HSF2, which is sensitive to elevated temperature, gets inactivated during high temperature stress, preventing interference with HSF1 activity in stressed cells (90). It is noteworthy to mention that the MAPK signalling network comprises several interconnected pathways, including extracellular signal-regulated kinases (ERKs), c-Jun N-terminal kinases (JNKs), and p38 MAPKs, which act as key sensors of environmental and cellular stress. Rather than functioning solely as activators of HSF1, these pathways can either enhance or attenuate HSF1 activity depending on the nature, intensity, and duration of the stress stimulus. Through phosphorylation of specific regulatory residues, MAPKs fine-tune the magnitude and duration of Hsp70 expression, thereby enabling cells to mount an appropriate stress response while avoiding high metabolic costs (91–94). In fish, activation of MAPK pathways has been reported following thermal stress, hypoxia, oxidative stress, salinity fluctuations, and pathogen exposure, demonstrating that Hsp70 regulation is integrated within broader cellular signalling networks involved in stress adaptation and immunity (95–98). In addition to transcriptional regulation, both HSF1 and Hsp70 are subject to extensive post-translational modifications that influence their biological activity. Phosphorylation, acetylation, Sumoylation, and ubiquitination have been shown to regulate HSF1 activation, trimerization, nuclear localisation, DNA-binding affinity, and transcriptional activity. Similarly, post-translational modifications of Hsp70 can alter its chaperone efficiency, substrate-binding properties, intracellular localisation, and interactions with co-chaperones and signalling proteins (99–102). These regulatory mechanisms contribute to tissue-specific and context-dependent stress responses, allowing different organs to tailor Hsp70 expression according to their physiological functions and environmental exposure.

Beyond classical transcription factor-mediated regulation, increasing evidence indicates that Hsp70 expression is also influenced by epigenetic mechanisms. DNA methylation, histone modifications, chromatin remodelling, and non-coding RNAs, particularly microRNAs (miRNAs), can alter the accessibility and transcriptional activity of heat shock genes (103–106). In aquatic organisms, environmental stressors such as temperature fluctuations, hypoxia, pollutants, nutritional status, and pathogen exposure have been associated with epigenetic modifications that influence Hsp expression profiles (107–109). These mechanisms may contribute to phenotypic plasticity and long-term adaptation by enabling organisms to modify stress-response pathways without altering the underlying DNA sequence. From an aquaculture perspective, understanding epigenetic regulation of Hsp70 may open new opportunities for nutritional and environmental interventions aimed at improving resilience, health, and welfare.

In invertebrates, insights into HSF regulation were obtained using the fruit fly *Drosophila*, in which only one HSF [i.e., HSF1] was identified to govern the heat shock response (80, 110–112). Although *Drosophila* has provided valuable mechanistic insights into HSF biology, caution is required when extrapolating findings broadly across aquaculture taxa because substantial differences exist in genome organisation, physiology, environmental adaptation, and life-history strategies among invertebrates and vertebrates. Therefore, evidence from commercially important aquaculture species is essential for understanding the practical significance of HSF–Hsp70 regulation under production conditions. Subsequently, many studies have extended these findings to a range of invertebrate (aquaculture) models, highlighting the conserved nature of HSF-mediated stress regulation (107–109). For instance, HSF1 isolated from the economically important penaeid shrimp showed a high degree of similarity with the insect HSF family and is widely expressed in different tissues, where it plays a significant role in Hsp70 regulation and enhancing stress resistance (107). These findings are particularly relevant for shrimp aquaculture, where environmental instability and disease outbreaks frequently compromise animal welfare and production efficiency.

Evidence from commercially important finfish species further supports the central role of HSF–Hsp70 signalling in stress adaptation. In Atlantic salmon (*Salmo salar*), rainbow trout (*Oncorhynchus mykiss*), Nile tilapia (*Oreochromis niloticus*), common carp (*Cyprinus carpio*), and zebrafish (*Danio rerio*), exposure to thermal stress, hypoxia, salinity fluctuations, and infectious agents has consistently been associated with Hsf1 activation and increased Hsp70 expression in tissues such as gills, liver, intestine, kidney, and immune organs (59, 113–115). Similar regulatory responses have been reported in molluscan aquaculture species, including oysters and mussels, where Hsp70 contributes to tolerance against thermal stress, pollutants, hypoxia, and pathogen challenges (116–118). Collectively, these studies demonstrate that while species-specific differences exist in the regulation and magnitude of Hsp70 responses, the Hsf–Hsp70 axis represents a broadly conserved mechanism underpinning stress resilience across diverse aquaculture taxa.

Further evidence for the key role of HSF1 in stress resilience and survival has been demonstrated in brine shrimp *Artemia franciscana*, live feed commonly used in the larviculture of fish and shrimp. Knockdown of HSF1 in the cysts of *A. franciscana* caused a significant reduction in their stress tolerance level compared to controls. Moreover, cysts that lack HSF1 showed reduced levels of three small heat shock proteins [sHsps]: p26, ArHsp21, and ArHsp22, as well as artemin, which is a homologue of ferritin. This indicates that the expression of their genes and optimal stress tolerance depend on HSF1. In addition, knockdown of HSF1 in *Artemia* nauplii led to premature mortality, highlighting the importance of HSF1-mediated Hsp expression in larval survival (119). From an aquaculture perspective, these findings highlight the relevance of HSF–Hsp70 regulatory pathways for larval robustness, survival, and welfare, particularly during vulnerable developmental stages.

Collectively, these studies demonstrate that the molecular regulation of Hsp70 through HSFs is not only fundamental to cellular stress responses but also plays a key role in shaping health and welfare outcomes in organisms. By modulating the ability of aquatic animals to respond rapidly and effectively to environmental and pathogenic stressors, HSF–Hsp70 signalling networks represent promising targets for resilience-based management strategies. Targeting these networks offers a promising avenue to improve health, maintain functional performance, and support animal welfare in the light of farming intensification and climate-driven pressures.

## 4 Hsp70 and acquired tolerance

When an organism encounters environmental or husbandry-related stressors, coordinated stress responses are activated at both physiological and molecular levels to restore homeostasis. The physiological stress response is characterized by the rapid release of stress hormones, such as catecholamines and cortisol, into the circulation. These hormones activate several metabolic pathways that cause mobilization of energy reserves, enabling the organism to cope with the stress (91, 92). In parallel, the cellular stress response, as described above, involves the production of a conserved set of Hsps, including Hsp70 (93–97).

Accumulating evidence suggests that the physiological and cellular stress responses are closely interconnected. Crosstalk between stress hormones and Hsps has been documented during stress episodes in organisms, with stress hormones shown to regulate the expression of several Hsp families, including Hsp27, Hsp70 or Hsp90 (98). Depending on the intensity, duration and context of the stressor, stress hormones can either upregulate or downregulate the expression of Hsps (98). Conversely, Hsps can modulate the activity of stress hormone receptors, thereby influencing downstream stress-response pathways (99, 100). This bidirectional interaction allows cells to cope with stress and maintain homeostasis under challenging conditions and is particularly relevant in aquaculture, where animals are frequently exposed to fluctuating environmental and husbandry-related stressors.

One of the most important functional attributes of Hsp70 is its involvement in acquired tolerance, also referred to as cross-tolerance. This phenomenon, observed across a wide range of organisms including mammals, birds, finfish, and shellfish, describes the capacity of prior exposure to a mild, non-lethal stressor to enhance resistance to a subsequent stress challenge of similar or different nature (101–106, 120–124). Acquired tolerance has been extensively studied in the context of thermal stress, where early work demonstrated that mammalian cells preconditioned with sublethal heat exposure exhibited increased tolerance to subsequent heat or chemical stress (125). Although the underlying mechanisms were initially unclear, later studies consistently linked this enhanced stress resistance to the induction of Hsp70 (126–128). In aquatic animals, exposure to mild or non-lethal heat stress has been shown to robustly induce Hsp70 expression and confer increased tolerance not only to elevated temperatures but also to a wide spectrum of abiotic and biotic stressors. These include heavy metals such as cadmium and zinc (93, 101), osmotic stress (129), acid exposure (130), ammonia toxicity (93), and infection stress caused by pathogenic microorganisms (127, 128, 131, 132). Heat shock–induced thermotolerance has been demonstrated in diverse taxa, including bacteria, bivalves, insects, and echinoderms (133–136), underscoring the conserved nature of this protective response.

Hsp70-mediated acquired tolerance should not be interpreted as universally beneficial or cost-free. The induction of Hsp70 requires substantial cellular energy and may divert resources away from growth, reproduction, immune investment and other performance-related processes. Repeated stress exposure or chronic Hsp70 induction may therefore impose metabolic costs, increase oxidative imbalance and compromise long-term production performance, particularly when animals are already exposed to suboptimal farming conditions (56, 137, 138).

This trade-off is especially important when considering the induction of Hsp70 using inducers as a management strategy. While controlled preconditioning or nutraceutical-based induction of Hsp may enhance short-term robustness, excessive or prolonged activation of the heat shock response could increase allostatic load and reduce growth efficiency, feed utilisation or welfare. Evidence from fish and other aquatic species suggests that stress-induced Hsp70 expression is often associated with elevated energy demand and altered antioxidant status, suggesting that the timing, dose, duration and physiological context of Hsp70 induction must be carefully optimised (69, 139, 140).

From a health and welfare management perspective, the ability to build robustness through controlled stimulation of Hsp70 has significant implications for aquaculture systems operating under conditions of increasing farming intensity and climate change. By strengthening the intrinsic ability of aquatic animals to withstand environmental fluctuations and pathogenic challenges, Hsp70-associated responses may reduce disease susceptibility and improve overall robustness without reliance on unsustainable therapeutic interventions. However, their practical use must be evaluated alongside long-term outcomes such as growth, metabolic burden, oxidative balance, immune competence, product quality and welfare. Advancing our understanding of how Hsp70-mediated tolerance can be harnessed through controlled stress conditioning, nutritional modulations or natural Hsp inducers, therefore, represents a promising approach for integrated health and welfare management in aquaculture.

## 5 Hsp70s and the immune responses

Hsp70 was initially considered a short-term cellular defence mechanism with essential housekeeping and cytoprotective roles (141). However, growing evidence over the past few decades reveals a much broader function, particularly in the regulation of the immune responses in both vertebrates and invertebrates (129, 130, 142–147). This dual role in stress mitigation and immune modulation positions Hsp70 as a key molecular interface linking cellular homeostasis to organismal health and welfare.

In vertebrates, immune defense depends on the coordinated action of both the innate and adaptive immune systems. In contrast, invertebrates primarily depend on the innate immune system as the first line of defence against invading pathogens. The innate immune system includes physical, cellular, and humoral factors that work together to recognize and neutralize infectious pathogens. In vertebrates, the antigen-specific adaptive immune system is triggered when the innate immune system is unable to effectively eliminate a pathogen (148). Several immune cells, including mast cells, neutrophils, monocytes, macrophages, dendritic cells, and natural killer cells, play key roles in both innate and adaptive immune responses (149). Central to immune activation are the pattern recognition receptors [PRRs], which detect conserved molecular signals associated with infection or tissue damage and initiate downstream immune responses (149–151). Several families of PRRs, including Toll-like receptors [TLRs], nucleotide-binding oligomerization domain–like receptors (NLRs), and retinoic acid–inducible gene–like receptors (RLRs), have been identified across different species. These receptors recognize two types of molecular patterns: pathogen-associated molecular patterns [PAMPs] and danger-associated molecular patterns [DAMPs] (149, 152). PAMPs are microbial-specific molecules, typically found in cell wall components, such as lipopolysaccharides, peptidoglycan, β-glucan, and flagellar components (69, 138). In contrast, DAMPs are components of damaged or apoptotic cells that can be derived from any cell compartments, including the nucleus and cytoplasm. DAMPs include both protein and non-protein molecules, with Hsp70s considered as prominent protein-associated DAMPs (149, 153).

Hsp70s act as endogenous immunomodulators capable of influencing both innate and adaptive immune responses. Under stress conditions, Hsp70s may be released into the extracellular environment through either active or passive mechanisms. Active release of Hsp70 occurs through extracellular vesicles, such as exosomes and ectosomes, particularly during immune cell-mediated cytotoxicity or viral infection. On the other hand, the passive release of Hsps occurs through cell death and subsequent lysis (154–157). Once extracellular, Hsp70s can interact with immune cells and influence immune signalling pathways, although the precise molecular mechanisms underlying these interactions remain incompletely known. Nevertheless, in mammalian models, it has been reported that antigen-presenting cells [APCs], such as dendritic cells [DCs] and monocytes, play a key role. These cells can internalise Hsp70-peptide complex spontaneously by receptor-mediated endocytosis or a receptor-independent mechanism (158), subsequently directing associated antigens into intracellular processing routes for presentation via major histocompatibility complex molecules for subsequent activation of T cells (149, 154, 159). Although these mechanisms are well documented in mammals, evidence supporting identical pathways in aquatic animals remains limited. Teleost fish possess functional antigen-presenting cells and major histocompatibility complex pathways, suggesting that some aspects of Hsp70-mediated antigen processing may be conserved (160–162). However, the extent to which fish utilise Hsp70-peptide complexes in antigen presentation remains insufficiently characterised, while crustaceans lack an adaptive immune system and therefore do not possess comparable MHC-dependent antigen-presentation pathways. It is likely that functionally analogous pathways operate in fish and crustaceans as well. In fact, some emerging evidence suggests that Hsp70 contributes to immune regulation in these species by enhancing pathogen recognition, supporting immune cell activation, and improving the efficiency of host defense responses. For example, genome-wide expression analyses in the crayfish *Procambarus clarkii* revealed that Hsp70 genes are activated under heat stress and bacterial challenge, with silencing of Hsp70 resulting in reduced expression of Toll-like receptor pathway genes, highlighting the functional connection between Hsp70 and immune response in aquatic crustaceans (163).

Importantly, the immunological effects of Hsp70 appear to be highly context dependent. While moderate and transient increases in Hsp70 expression are frequently associated with enhanced resistance to environmental stress and infectious challenges, excessive, prolonged, or uncontrolled Hsp70 induction may alter immune homeostasis and, in some cases, promote inflammatory responses (164–166). The biological outcome depends on several factors, including the magnitude and duration of Hsp70 induction, tissue specificity, developmental stage, environmental conditions, and the nature of the immune challenge. Consequently, statements that Hsp70 universally enhances immune competence should be interpreted with caution, as both immunostimulatory and immunomodulatory effects have been reported across different species and experimental settings (26, 167, 168).

Evidence from aquaculture species supports this context-dependent role. In fish, elevated Hsp70 expression has been associated with improved resistance to bacterial, viral, and environmental stress challenges, often through enhanced innate immune functions and cellular protection (169–172). However, the benefits are not always proportional to the level of Hsp70 induction, indicating that optimal rather than maximal activation of Hsp70-associated pathways is likely to be most beneficial for health and welfare management.

Collectively, these findings highlight the importance of elucidating Hsp70-mediated immune mechanisms in farmed aquatic vertebrates and invertebrates. A deeper understanding of how Hsp70 integrates stress responses with immune regulation may reveal critical protective pathways that can be harnessed to build resilience against diseases and support sustainable health and welfare management in farmed aquatic animals.

## 6 Eukaryotic Hsp70s and disease prevention in aquatic animals

As mentioned above, Hsp70s are ubiquitous across prokaryotic and eukaryotic organisms. In mammalian and other vertebrate systems, eukaryotic Hsps, particularly Hsp70, are well documented to elicit immune responses and enhance resistance to infection, thereby influencing disease outcomes (160–162, 173–178). In aquaculture animals, increasing evidence similarly associates Hsp70 induction with enhanced robustness against both pathogens and environmental stress, supporting the view that Hsp70 is a vital biomolecule that can play a key role in preventive health management (179–184). However, much of the available evidence originates from laboratory-based studies conducted under controlled experimental conditions, and relatively few studies have validated Hsp70-mediated protection under commercial aquaculture settings. Early experimental evidence in aquaculture organisms came from the work of Sung and colleagues (185), who used non-lethal heat shock [NLHS] as a classical stimulus to trigger the induction of eukaryotic stress proteins, including Hsp70. Using crustacean brine shrimp *Artemia* as a biological organism, the authors showed that a short exposure to NLHS, followed by a recovery, could confer cross-protection to *Artemia* against infection stress caused by pathogenic vibrios, such as *Vibrio campbellii* or *Vibrio proteolyticu*s. The increased survival of the NLHS-exposed larvae, in comparison to unshocked controls, was attributed to the production of Hsp70 in the shrimp. Follow-up studies in *Artemia* reported that NLHS-induced Hsp70 also promoted tolerance to abiotic stress (e.g., thermal and salinity stress), consistent with a broader cross-tolerance phenotype (186). When an organism is exposed to a stress inducer like NLHS, a variety of Hsps, rather than only Hsp70, are induced. This raises questions about the functional role of Hsp70 in the observed traits of cross-protection. Subsequent functional research was therefore conducted to address this question (181). The results suggested that RNAi-mediated depletion of Hsp70 reduced the survival of *Artemia* under heat shock and following *V. campbellii* challenge. In comparison to *Artemia* nauplii, which contained Hsp70, mutant *Artemia* that lacked Hsp70 had a 41% decrease in survival when subjected to heat stress and a 34% decrease in survival when infected with *V. campbellii*. This clearly suggests that Hsp70 plays a primary role in contributing to robustness traits in organisms. Although *Artemia* provides a powerful experimental system for investigating fundamental mechanisms of stress adaptation and disease resistance, translational studies are warranted to extrapolate findings directly to commercially farmed species, as differences in physiology, life history, production environments, and management practices may influence the magnitude and persistence of Hsp70-mediated protection.

Evidence for Hsp70-associated protection has also accumulated in commercially important crustaceans under laboratory settings. For example, in farmed penaeid shrimp, *P. vannamei*, NLHS regimes (e.g., short exposure to a heat shock of 38 °C for 30 min or chronic exposure to a temperature of 38 °C for 5 min every day for 7 days) were shown to elevate Hsp70 and Hsp90 and improve the tolerance responses of the shrimp towards AHPND-causing *V. parahaemolyticus* challenge (127). A subsequent study conducted on the same shrimp species showed that NLHS-mediated Hsp70, by short exposure to 38 °C for 1 h followed by 8 h recovery, also conferred protection to *P. vannamei* post larvae against stress caused by heat, ammonia, and heavy metals (93). Beyond these priming studies, more mechanistic research has reinforced the importance of Hsp70 in building resilience towards diseases. For example, RNAi knockdown of Hsp70 in *P. vannamei* markedly compromised animal condition, resulting in reduced activity and feed intake, hepatopancreatic tissue damage, and increased mortality. During *V. parahaemolyticus* infection, shrimp with silenced Hsp70 exhibited increased *Vibrio* abundance in the intestine and exacerbated tissue damage, accompanied by broad downregulation of immune-related genes, including antimicrobial peptides, cytokines, and components of the prophenoloxidase system. These results highlight a broader role for Hsp70 in maintaining host homeostasis during infection (187). Collectively, these findings are particularly relevant for health and welfare management because they connect Hsp70 status to functional outcomes that are crucial for productivity in aquaculture production systems. Nevertheless, the practical implementation of NLHS protocols in commercial aquaculture may be constrained by operational, economic, and animal welfare considerations. For example, repeated exposure to elevated temperatures may be difficult to standardise across production systems and may not be appropriate for all species, developmental stages, or farming environments. Therefore, the value of these studies lies primarily in demonstrating the protective potential of Hsp70 induction rather than advocating NLHS as a universally applicable management tool.

Comparable observations have been documented in other farmed aquatic organisms. For example, in the California native oyster *Ostreola conchaphila*, increased thermotolerance has been correlated with the upregulation of constitutive and inducible Hsp70 (188). In another study, juveniles northern bay scallops *Argopecten irradians irradians* exposed to a 3 h NLHS at a sublethal temperature of 32 °C acquired tolerance to a lethal heat treatment at 35 °C that lasted for at least 7 days, a response directly correlated with increased Hsp70 levels (169). This inducible Hsp70-mediated cross-tolerance trait was not limited to crustaceans but also occurs in finfish. For example, a short 30 min NLHS at 38 °C followed by a 8 h recovery period at 28 °C of the common carp *Cyprinus carpio* juveniles was reported to enhance subsequent survival at lethal temperatures, coinciding with elevated Hsp70 levels in gill tissue (131). Similarly, an NLHS protocol from 12 to 25 °C for 1 h with a subsequent recovery for up to 10 days induced thermotolerance in Coho salmon, with the effect prolonged for about a week after NLHS (170). In this study, the increased thermotolerance was associated with an increased level of Hsc70 in multiple tissues. Collectively, these studies indicate that Hsp70 is frequently associated with enhanced tolerance to environmental and pathogenic stressors across diverse aquatic taxa. However, the magnitude, duration, and biological significance of this protection vary among species, life stages, and stress contexts, highlighting the need for species-specific validation.

The induction of Hsp70 and perhaps other members of the Hsp families following an NLHS may well protect farmed crustaceans and fishes in similar ways, rendering them more resistant against pathogenic and/or abiotic stressors. At present, however, evidence primarily supports a contributory role for Hsp70 within a broader network of stress-response mechanisms rather than identifying Hsp70 as the sole determinant of disease resistance. Future studies should therefore focus on validating Hsp70-based interventions under aquaculture settings and identifying practical approaches, such as non-invasive, natural Hsp inducers, that can stimulate protective responses without imposing additional management burdens. Available data support the view that eukaryotic Hsp70s are important mediators of cross-tolerance in aquatic animals. While these findings highlight the potential of Hsp70-based strategies for improving resilience, further research is needed to establish their scalability, consistency, and long-term effectiveness under commercial aquaculture conditions.

## 7 Microbial Hsp70 and disease prevention in aquaculture animals

In addition to endogenous Hsp70s, heat shock proteins of microbial origin have attracted increasing attention as immunologically active molecules in aquaculture species. Prokaryotic Hsp70s (commonly referred to as DnaK), derived from bacteria and yeasts, exhibit high immunogenicity that are comparable to eukaryotic Hsp70s. A growing number of experimental studies indicate that microbial Hsp70s can stimulate protective immune responses in both finfish and shellfish, supporting their potential application in disease prevention strategies such as vaccination and dietary immunostimulation (170–172, 189). Several studies in finfish have demonstrated the vaccine potential of recombinant microbial Hsps against infectious pathogens. For example, recombinant Hsp90, Hsp33, Hsp70, and DnaJ derived from *Photobacterium damselae* subsp. *Piscicida* were examined as vaccine antigens in Asian seabass in combating photobacteriosis, a bacterial septicaemia caused by *P. damselae*. All recombinant proteins conferred measurable protection, with recombinant forms of Hsp90 [rHsp90], rHsp33, rHsp70, and rDnaJ exhibiting a relative increase in survival percentage of 48.3, 62.1, 51.7, and 31.0%, respectively, when subjected to challenge with the bacterial pathogen. Among the different groups, those that received rHsp33 demonstrated high expression levels of immune-related genes, elevated antibody titres, and enhanced bactericidal activity against *P. damselae*. The groups immunised with rHsp70 also showed a high level of immune response against *P. damselae* infection. These findings indicate that microbial Hsps can function as effective immunogens, although their protective efficacy appears to vary depending on the specific Hsp family member and host–pathogen combination (189). Similar findings have been reported for DNA vaccines prepared using bacterial Hsp70 [DnaK] and GroEL [Hsp60 bacterial homolog], which boost the immune response and confer protection against bacterial nocardiosis in hybrid snakehead [*Channa argus* male x *Channa maculata* female] (172). In parasitic disease models, immunisation with rHsp70 from *Cryptocaryon irritans* significantly increased resistance in spotted grouper Epinephelus *coioides*, further supporting the broad applicability of microbial Hsp70-based vaccination approaches (171).

Beyond vaccination, microbial Hsp70s have also been explored as oral immunostimulants, particularly in farmed invertebrates, which lack the adaptive immune system and rely only on the innate immunity to fight infection. A series of pioneering studies conducted using the *Artemia-Vibrio* host-pathogen model system demonstrated that feeding of recombinant DnaK significantly increased resistance of *Artemia* larvae to *Vibrio campbellii* infection (190). A subsequent investigation by Baruah et al. (191) linked this enhanced resistance to increased phenoloxidase activity, a key component of the innate immune system in crustaceans, suggesting that microbial Hsp70s can prime the innate immune system (192, 193). A similar protective effect was observed in *P. vannamei*, where feeding shrimp with a diet supplemented with non-pathogenic *Escherichia coli* overproducing DnaK markedly improved survival following *V. harveyi* challenge (180). In finfish European sea bass larvae, feeding of sodium alginate encapsulated recombinant DnaK has been shown to cause significant upregulation of a battery of immune genes [i.e., *cxcr4, cas1, hep* and *dic*], and enhance survival rates of the larvae upon challenge with *V. anguillarum* (194), highlighting the feasibility of microbial Hsp70 delivery through feed-based strategies in improving the health and welfare of the farmed species.

**Figure 2:**
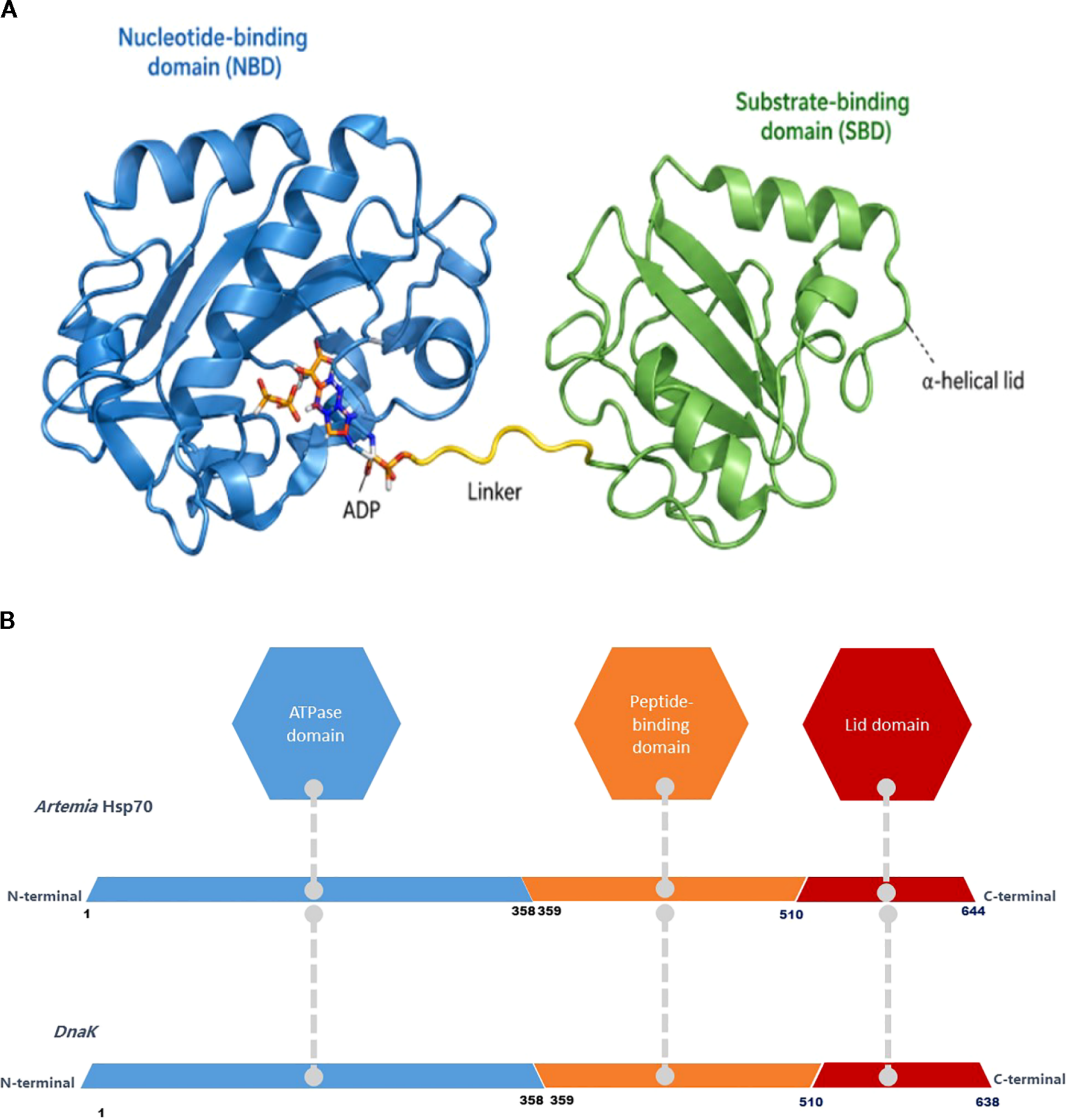
**(A)** Three-dimensional structure of Hsp70 showing the N-terminal nucleotide-binding domain (NBD) and the C-terminal substrate-binding domain (SBD), connected by a flexible linker. The SBD contains an α-helical lid that regulates substrate binding, while ADP is bound within the NBD. **(B)** Schematic comparison of the domain organisation of *Artemia* Hsp70 and bacterial DnaK proteins. Both proteins exhibit the conserved Hsp70 architecture consisting of an N-terminal ATPase domain, a peptide-binding domain, and a C-terminal lid domain. Domain boundaries are indicated by amino acid positions, highlighting the high structural conservation between *Artemia* Hsp70 (644 aa) and DnaK (638 aa).

At the molecular level, the genome of bacterial Hsp70 DnaK shares a high degree of structural conservation with eukaryotic Hsp70s, with approximately 60% sequence similarity. Baruah et al. (2012) compared two evolutionarily diverse Hsp70 proteins, one from *Artemia* and the other from *Escherichia coli* (DnaK), for their ability to protect *Artemia* against a *Vibrio* challenge. Both recombinant proteins, produced in *E. coli* and fed to *Artemia* nauplii prior to *Vibrio* challenge, conferred comparable levels of protection against the *Vibrio* challenge, despite their taxonomic divergence (2012). Amino acid sequence alignment showed approximately 59.6% identity, particularly in the peptide-binding domain, which is considered the putative innate immunity-activating portion. This suggests that the protective function of the recombinant Hsp70 proteins against *Vibrio* likely resides within this conserved domain. Baruah and colleagues (172) further studied the structural basis of Hsp70-mediated protection in *Artemia* by examining specifically the role of the peptide-binding domain during *Vibrio* challenge (**Figure 2A**). Using recombinant truncated variants of endogenous *Artemia* Hsp70 and bacterial DnaK that retained the peptide-binding region, the authors showed that these fragments conferred significant protection against *V. campbellii*. Notably, the level of protection was comparable to that observed with full-length recombinant Hsp70 or DnaK (195, **Figure 2B**), suggesting that the ATPase domain of *Artemia* Hsp70 or DnaK protein activity is not essential for the protective effect and that the peptide-binding domain is a key determinant of Hsp70-driven immune protection against bacterial infection.

From an application perspective, recombinant Hsp70-based products may have favourable prospects for commercial development because regulatory frameworks for recombinant proteins are already well established in both human and veterinary medicine. Nevertheless, approval requirements for recombinant Hsp70 products intended for aquaculture applications will depend on their mode of use (e.g., vaccine, feed additive, or immunostimulant), production platform, environmental safety, and food-safety considerations. While further validation under commercial conditions is required, the existing regulatory experience with recombinant protein-based products suggests that Hsp70-derived interventions may have a feasible pathway towards practical implementation in aquaculture disease management (196–198).

## 8 Inducers and co-inducers of Hsp70 as tools for health and welfare management in aquaculture animals

Experimental studies in aquaculture animals have consistently shown the protective potential of Hsp70s in mitigating susceptibility to diseases and stress-related damage. However, translating these findings into practical applications requires effective and welfare-compatible strategies to either induce endogenous Hsp70 production or precisely deliver functional Hsp70 to the target organism. The most commonly used experimental approach to induce Hsp70 has been non-lethal heat shock (NLHS), owing to its reliability in activating the heat shock response at the cellular and organismal level. Despite its effectiveness under controlled conditions, NLHS is poorly suited for commercial aquaculture, as abrupt temperature fluctuations can disrupt physiological homeostasis, compromise welfare, and lead to elevated mortality in cultured animals (199). Consequently, alternative, non-invasive methods for modulating Hsp70 expression are needed.

**Figure 3:**
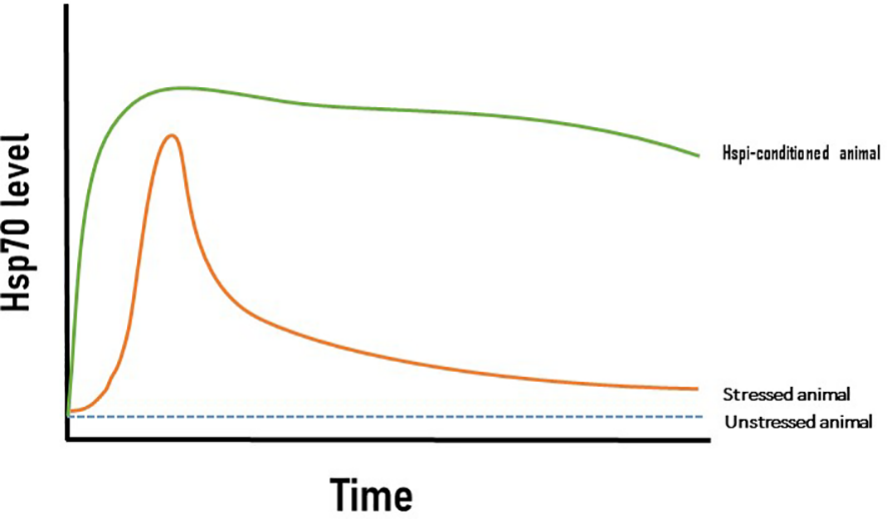
Simulation of Hsp70 production level in unstressed organisms [control], stressed organisms, and organisms exposed to a Hsp70-inducing compound. Upon exposure to such a compound, there is an increase in the synthesis of inducible Hsp70 within minutes, which remains elevated for a long period of time following exposure to a stressor. Animals exposed to an environmental or pathogen stress also produced high levels of inducible Hsp70. However, a large quantity of Hsp70 is utilised in the repair of damaged cells. Hence, the level decreased relatively fast. The quantity of Hsp70 utilized for tissue repair purposes is associated with the rate of response. A quick rate of repair results in the reduced utilization of Hsp70, and hence a prolonged response.

To date, a wide range of compounds has been identified that can influence Hsp70 regulation. Based on their mode of action, these compounds are generally classified as Hsp inducers or Hsp co-inducers. Hsp inducer directly activates heat shock transcription factors, primarily HSF1, thereby triggering Hsp expression independently. In contrast, Hsp co-inducer does not initiate Hsp expression on its own but potentiates Hsp induction in the presence of mild physiological or environmental stressors (**Figure 3**). This distinction is particularly relevant from a welfare perspective, as co-inducers may allow fine-tuning of the stress response without eliciting excessive cellular stress.

Many researchers have been working to identify novel and natural Hsp-inducing compounds, especially those derived from plant and microbial sources. Such compounds offer the dual advantage of supporting animal health and resilience while aligning with consumer preferences for environmentally friendly and chemical-free production systems. Over the past two decades, numerous studies have investigated the functional relevance of Hsp inducers and co-inducers in enhancing stress tolerance, immune competence, and disease resistance in aquaculture animals. In this review, particular emphasis is placed on naturally derived Hsp inducers and Hsp co-inducers (**Table 1**), highlighting their potential as practical, nature-based tools for integrating Hsp70 modulation into health and welfare management strategies for sustainable aquaculture.

**Table 1:** Inducers and co-inducers of heat shock proteins in aquaculture animals from plant and microbial origin.

Hsp inducers	Aquatic organism	Stressor (biotic and/or abiotic)	Conditioning approach	Phenotype recorded		Induced Hsp	References
Plant-based Hsp inducers							
Cactus extract	*P. scalare*	Ammonia (LC50 - 1.1 mg/L for 24 h)	Pretreatment	Induced *hsp70* and *hsp90* in liver, muscle and gill		*hsp70* mRNA *hsp90* mRNA	(40, 200)
	*A. franciscana*	Thermal (41 °C for 20 min) Hypersaline (100 g/L for 36 h)	Pretreatment (OD-152 ppb/SOD-76 ppb for 1h + NLHS (37 °C/30 min + 6 h recovery)	Maximum protection against thermal and hypersaline stresses		*hsp70* mRNA Hsp70 (protein)	(186)
	*C. carpio* (fingerlings)	Ammonia (LD50 - 5.92 mg/L/LCT (total mortality) - 14.21 mg/L for 48 h)	Pretreatment (2 μl/50L for 2 h)	Survival increased by 20% Induced Hsp70 in gill and muscle		Hsp70 (protein)	(201)
	*A. franciscana*	*V. campbellii* *V. harveyi* (10^7^ cells/mL for 48 h)	Pretreatment (OD-5 mg/L for 2 h)	Maximum protection against the vibrios-induced expression of hsp70, *proPO*, and *tgase*, high prooxidant capacity		*hsp70* mRNA Hsp70 (protein)	(130)
	*Danio rerio*	Thermal (37 0C for 1 h)	Pretreatment (for 4 h at 28.5 0C)	Induced *hsp70* expression		*hsp70* mRNA	(202)
	*C. carpio*	Confinement (5 fish/14,200 cm3 for 1 h)	Pretreatment (24 h)	Induced *hsp70* expression		*hsp70* mRNA	
	*A. franciscana*	*V. campbellii* (10^7^ cells/mL for 36 h)	Pretreatment (OD-160 μl/L for 1 hr)	Improved protection against *V. campbellii* Increased virulence factors production		DnaK (Hsp70) in *Vibrio*	(203)
	*Acipenser persicus* fingerlings	*A. hydrophila* (100 µl IP injection 3/7 days post-injection 3 × 10^5^ cfu/mL)	Pretreatment (OD-100 mg/L for 2h)/pretreatment + challenge with Aeromonas (IP injection)	Induced *hsp70* expression		*hsp70* mRNA	(204)
Pyrogallol	*A. franciscana*	*V. harveyi* (10^7^ cells/mL for 48 days)	Pretreatment (OD-595 µM for 2 h)	Maximum protection against *V. harveyi* *hsp70*, *proPO*, and tgase genes expression, high prooxidant capacity		*hsp70* mRNA *Hsp70* (protein)	(205)
Phloroglucinol	*A. franciscana* *M. rosenbergii*	*V. parahaemolyticus* (10^7^ cells/mL for 48 h)	Pretreatment of *Artemia* (OD-30 µM) and *Macrobrachium* (OD-5 µM) for 2 h	Maximum protection against *V. parahaemolyticus* h*sp70* knockdown reduces the tolerance against *Vibrio*		*hsp70* mRNA Hsp70 (protein)	(103)
	*A. franciscana*	*V. parahaemolyticus*, *V. harveyii* (10^7^ cells/mL for 12 h)	Continuous exposure (2 µM for 60 h)	Maximum protection against vibrios, induced *hsp90*, *hsp70*, and *proPO*, immune gene expression		*hsp70* mRNA *hsp90* mRNA	(206)
	*A. franciscana*	*V. parahaemolyticus* (10^7^ cells/mL for 6 h)	Pretreatment (OD-2 μM)	*hsp70*, and *hsp90* only showed significantly elevated expression		*hsp70* mRNA *hsp90* mRNA	
Carvacrol	*A. franciscana*	Thermal stress (41 °C/20 min), *V. harveyii* (10^7^ cells/mL for 48 h)	Pretreatment (OD-66.4 μM for 2 h)	Increased survival against thermal stress and *V. harveyi*		*hsp72* mRNA	(207)
	*O. niloticus*	No stress applied	Diets incorporate with oregano essential oil (highly rich in carvacrol) at 0, 0.25, 0.5, and 1 g/kg for eight weeks (T0 = 32 °C)	Relieved heat stress Reduced relative expression of *hsp70*		*hsp70* mRNA	(208)
Curcumin	*Puntius sophore*	Sub-lethal heat shock (36 °C for 6 h)	Supplement (1.5% for 60 days)	Induced hsps genes expression in gills and the liver		*hsp70* mRNA *hsp90* mRNA *hsp110* mRNA	(209)
	*O.niloticus*	Cold stress (Avg. 9.0 ± 0.5 °C)	Incorporate into the diet (1-3% for 5 weeks) and CC5 gene expression	Improved weight gain, FCE, SGR, CF, antioxidant capacity, and induced *hsp70*, IL-1β,		hsp70 mRNA	(210)
Turmeric oil	*Pangasianodon hypophthalmus* fingerlings	*Ichthyophthirius multifiliis* associated with *Aeromonas hydrophila* (10^2^–10^7^ cfu/mL	Supplement (10 ppm for 120 h)	Significantly induced the expression (2-fold) in both *hsp70* and *hsp90*		*hsp70* mRNA, *hsp90* mRNA	(211)
Microalgae (Mixture)	*A. franciscana*	No stress applied	Microalgae (1–2 x 10^6^ cells/mL), including *C. vulgaris*, *M. gracile*, and *P. kessleri*,	Induced *hsp70* expression		*hsp70* mRNA	(31)
*Chlorella vulgaris*	*O. niloticus*	Chronic chlorpyrifos exposure (15 mg/L 4–8 wks)	Diets incorporate (1, 2, and 3% for 4–8 weeks)	Induced hepatic *hsp70*, GPx, and GSS, and decreased GSR, increased mRNA levels of IL-1β, TNF-α, TGFβ1, and IL-8 in the spleen and head kidney after CPF exposure		*hsp70* mRNA	(212)
*Nannochloropsis oculata*	*Cromileptes altivelis*	Nervous Necrosis Viral (NNV)	Oral applies (376 µL for 23 days)	Induced production of *hsp70* in brain tissue and blood cells after challenge		*hsp70*mRNA	(213)
*Gracilaria tenuistipitata*	*Litopenaeus vannamei juveniles*	Vibrio harveyii (1x10^6^ cfu/mL)	Diet incorporation (0.5, 1.0, and 2.0 g/kg for 16 weeks)	Induced *hsp70* expression		*hsp70* mRNA	(214)
*Pandanus tectorius* fruit extract	*P. vannamei*	*V. parahaemolyticus* (LD50 = 10^6^ cells/mL for 24h)	Pretreatment (OD-6 g/L for 24 h)	Increased survival challenges with *V. parahaemolyticus* and immune-related proteins; *Hsp70*, *proPO*, peroxinectin, penaeidin, crustin, tgase		*hsp70* mRNA *Hsp70* (protein)	(184)
*Pandanus tectorius* leaf extract	*P. vannamei*	*V. parahaemolyticus* (LD50 = 10^6^ cells/mL for 24h)	Pretreatment (OD-6 g/L for 24 h)	Increased survival challenges with *V. parahaemolyticus* and expression of immune-related protein *Hsp70* and immune genes; proPO and crustin		*hsp70* mRNA *Hsp70* (protein)	(215)
*Pandanus tectorius* leaf extract	*C. carpio* (Avg. wt-12 g)	*Aeromonas hydrophila* (10^7^ cells/mL)	Diet incorporation (5 g/kg to 30 g/kg leaf extract for 8 weeks)	No significant expression of hsp70		Not induced *hsp70* mRNA	(216)
*Pandanus tectorius* leaf extract	*A. franciscana*	*V. campbellii* (10^6^ cfu/mL)	Pretreatment (OD = 1g/L for 2 h)	Induced *hsp70*, *hsp60*, *hsp90*		*hsp70* mRNA	(217)
Anthraquinone extract from *Rheum officinale*	*Megalobrama amblycephala* fingerlings	*Aeromonas hydrophila* (5x10^7^ cells/mL for 6, 12, 24, and 48 h)	Diet incorporation (0.1% Anthraquinone extract for 70 day)	Induced serum *hsp70* expression 6 h before and 6 h after infection		*hsp70* mRNA	(218)
*Forsythia suspensa*		*V. parahaemolyticus* (injection- 5 × 10^6^ cfu/mL)	Diet incorporation (0.01%–0.02% FSE for 60 days)	Induced *hsp70*		*hsp70* mRNA	(219)
Soybean isoflavones	*Trachinotus ovatus*	pH stress (9.2 for 24 h)	Incorporate feed (40–60 mg/kg)		Induced the hepatic *hsp70* and *hsp90* expression and SOD activity	*hsp70* mRNA, *hsp90* mRNA	(220)
Sodium ascorbate	*A. franciscana*	*V. harveyi* (10^7^ cells/mL for 48 h)	Pretreatment (OD-2000 ppm for 2h)		Maximum protection against *V. harveyi*, *hsp70* knockdown shows less survival	*hsp70* mRNA	(221)
Hawthorn extract	*Trachinotus ovatus*	*Vibrio harveyii*	Diet incorporation (0.5–6 g/kg for 8 weeks)		*hsp70* and *hsp90* downregulated	Downregulated *hsp70* mRNA *hsp90* mRNA	(222)
Essential oils from *M. alternifolia* and *L. citrata*	*A. franciscana*	*V. campbellii* (10^7^ cells/mL for 24 h)	Continuous exposure (OD- M. alternifolia (0.0008%) and *L. citrata* (0.002%)		two-fold higher expression of *hsp70* and tgase1	*hsp70* mRNA	(223)
Galactomannan oligosaccharides/garlic and labiatae essential oils	*Dicentrarchus labrax*	*V. anguillarum* 10^5^ cfu per fish (2h, 24 h, and 7 days post-infection	Diet incorporation (5000 ppm -galactomannan oligosaccharides/200 ppm - garlic and labiatae essential oils		Not induced *hsp70* or *hsp90* in head kidney	*hsp70* mRNA	(224)
Soybean oil	*Oncorhynchus mykiss*	No stress applied	Feed incorporation (8 weeks with 14% from the diet)		Induced muscle *hsp70*	*hsp70* mRNA	(225)
*Mentha piperita*	*Rutilus caspicus*	No stress applied	Feed incorporation (8 weeks with 4 g/kg from the diet)		Not induced *hsp70* in the intestine and kidney tissues	*hsp70* mRNA	(226)
Hesperidin	*Procambarus clarkii*	White spot syndrome virus	Diet incorporation (50–150 mg/kg for 8 weeks)		Induced *hsp70*	*hsp70* mRNA	(227)
Astaxanthin and emodin	*Pelteobagrus fulvidraco*	Crowding stress (150 g/L) and Proteus mirabilis (1x108 cfu/mL at 0–120 h)	Diet incorporation (60 days feeding with Astaxanthin 80 mg/kg, emodin 150 mg/kg, and combination)		Induced hepatic *hsp70*	*hsp70* mRNA	(228)
*Syzygium cumini* leaf extract	*Cyprinus carpio*	*Aeromonas hydrophila* (IP injection - 1 × 10^7^ cells/mL)	Diet incorporation (8 weeks with 15% from the diet)		Induced liver *hsp70*	*hsp70* mRNA	(228)
Blackberry syrup	*Oreochromis niloticus*	*Plesiomonas shigelloides* (3x10^8^ cfu/mL)	Diet incorporation (90 days with 15 g/kg from the diet)		Improved the protection, Induced spleen *hsp70*	*hsp70* mRNA	(229)
Microbial Hsp inducers							
β-glucan	*O. niloticus*	Stocking density (LD-200/m^3^, MD-400/m^3^ HD-600/m3)	β-glucan incorporated diet		HD induced the transcription of INF-γ, *hsp70*, LD upregulate TNF-α, IL-1β	*hsp70* mRNA	(36)
Poly-ß-hydroxybutyrate (PHB)	*A. franciscana*	*V. campbellii* (10^7^ cells/mL for 48 h)	Continuous exposure (100 mg/L)		Maximum protection against *V. campbelli*, induced *hsp70* expression	*hsp70* mRNA	(230)
	*P. monodon* PL	*V. campbellii* (10^6^ cells/mL for 12 h)	Enrich with Bacillus sp. (55% PHB/DW for 6 h)		Induced *proPO*, *tgase*, *hsp70* expression	*hsp70* mRNA	(231)
*Aspergillus oryzae* (ASP)	*O. niloticus*	Hypoxia (No aeration and less water volume)	ASP diet 1 × 10^6^/1 × 10^8^ cfu/g for 60 days)		Induced blood immune responses and TNF-α, IL-1β, and *hsp70* expression and also stimulate the oxidation status	*hsp70* mRNA	(36)
*Debaryomyces hansenii*	*Dicentrarchus labrax* larvae	No stress applied	Diet contains (4.3% w/w from 23 dph-48 dph)		No effect on *hsp70* expression	*hsp70* mRNA	(232)
	*Mycteroperca rosacea* juveniles	*Aeromonas hydrophila* (10^7^ cells/mL injection)	Diet incorporation (1.1% *D. hansenii* for 5 weeks)		Induced *hsp70* in liver and intestine	*hsp70* mRNA	(233)
*Mannan oligosaccharide*	*Carassius auratus gibelio*	*Aeromonas hydrophila* (10^8^ cells/mL injection)	Diet incorporation (480 mg/kg for 10 weeks)		Induced hepatic *hsp70* at 2 days and 7 days after infection	*hsp70* mRNA	(234)
*Lactobacillus pentosus*	*Anguilla japonica*	Edwardsiella tarda (0.1 ml dose of 3.5 × 10^8^ cfu/mL)	Diet incorporation (108 cfu/g for 4 weeks)		Induced *hsp70* in liver and intestine	*hsp70* mRNA	(235)
Immunogen® β-Glucan and mannaoligosaccharides (MOS) derived from yeast	*Oncorhynchus mykiss*	*Aeromonas hydrophila* (1x10^7^ cfu/mL)	Diet incorporation 2 g/kg Immunogen® for 45 days)		Downregulated *hsp70* in head kidney	*hsp70* mRNA	(236)

OD, Optimum dose; TAN, Total ammonia nitrogen.

### 8.1 Inducers of Hsps from plant sources

#### 8.1.1 Pyrogallol

Pyrogallol is a naturally occurring phenolic compound found in various fruits and vegetables and is known to exhibit a broad spectrum of health-promoting effects in experimental animals. Its health-promoting effect has generally been linked to antioxidant activity, particularly through scavenging of stress-induced free radicals (237, 238). Wang et al. (239) showed that pyrogallol induced antioxidant activity by increasing the activity of key antioxidant enzymes, superoxide dismutase, catalase, and peroxidase, in *Microcystis aeruginosa* algal cells following prolonged exposure.

Beyond its antioxidant effects, pyrogallol has also been identified as a modulator of the heat shock response. In *Artemia*, short-term pre-exposure to micromolar concentrations of pyrogallol induced Hsp70 expression and significantly improved resistance to subsequent bacterial challenge. This enhanced resistance was associated with activation of innate immune pathways, including the prophenoloxidase and transglutaminase systems, suggesting a functional link between Hsp70 induction and immune priming (71, 205). Mechanistic investigations indicated that Hsp70 induction was mediated through a transient prooxidant effect, likely involving the generation of hydrogen peroxide, rather than classical antioxidant activity (205).

Supporting evidence for the prooxidant action of pyrogallol comes from microbial models, where growth inhibition induced by pyrogallol exposure could be partially reversed by catalase treatment, confirming the involvement of hydrogen peroxide-mediated oxidative stress (240). These findings highlight the dual nature of pyrogallol, which can act as either a prooxidant or an antioxidant depending on whether the host/cells are subjected to pre-exposure or continuous exposure conditions. In aquaculture-relevant contexts, short-term exposure appears to favour controlled oxidative signalling that triggers protective stress responses, whereas prolonged exposure may exert antimicrobial or cytotoxic effects. To our knowledge, the first evidence indicating that pyrogallol could be used as an Hsp inducer to combat infection stress was on *Artemia*. Another research by Defoirdt et al. (241) indicated that continuous exposure of the freshwater prawn *Macrobrachium* larvae to pyrogallol improved survival of the prawn larvae challenged with *V. harveyi.* The authors ascribed the *Vibrio*-protective effect of the compound to its prooxidant action, but the potential involvement of Hsp70 was not explicitly examined in that study. Overall, while pyrogallol shows promise as an Hsp70-inducing compound with protective effects against infection stress, its practical relevance across aquaculture species remains largely unexplored. Further studies are needed to define safe exposure regimes, clarify mechanisms of action, and assess their applicability in farmed fish and shellfish.

#### 8.1.2 Phloroglucinol

Phloroglucinol [1, 3, 5 – trihydroxybenzene] is a naturally occurring phenolic compound abundantly found in plants, especially in brown algae, as well as in certain microbes, e.g., *Pseudomonas* spp. It exhibits a wide range of pharmacological actions, such as antiviral, antibacterial, antifungal, antihelminthic, and anti-stress activities (242, 243). Although the molecular mechanisms of these pharmacological functions of phloroglucinol are not yet fully understood, some evidence suggests that many of these effects are mediated, at least in part, through the induction of Hsp70. These inferences are made based on the findings from a series of *in vivo* studies. Experimental studies using the *Artemia–Vibrio* host–pathogen model have provided key insights into this mechanism. Short-term exposure of *Artemia* to phloroglucinol followed by a recovery period resulted in a marked upregulation of Hsp70, which was associated with enhanced resistance to *V. parahaemolyticus* infection and improved tolerance to thermal stress (103). Functional validation using RNAi revealed that suppression of Hsp70 neutralised the protective effects of phloroglucinol, highlighting that Hsp70 induction is a critical determinant of the observed resistance. Further analyses indicated that phloroglucinol increased lipid peroxidation levels, suggesting that its pro-oxidant activity may act as a trigger for Hsp70 induction (103), consistent with reports for other bioactive Hsp-inducing compounds (71).

Beyond immediate stress protection, phloroglucinol has also been shown to induce long-lasting, transgenerational resilience against biotic vibrios, and lethal abiotic stressors i.e., thermal and ammonia (206, 244). Early-life exposure of *Artemia* to phloroglucinol resulted in enhanced resistance to both thermal stress and *Vibrio* infection in subsequent generations, with protective effects persisting up to the third generation even in the absence of continued treatment (206). This inherited robustness was associated with sustained upregulation of innate immune genes and linked to epigenetic modifications, including DNA and RNA methylation and histone modification patterns, indicating that phloroglucinol can induce durable stress memory through epigenetic reprogramming (206).

Building on these findings, phloroglucinol has been evaluated as a nature-based health management tool in freshwater prawn (*Macrobrachium rosenbergii*) hatcheries, where vibriosis represents a major welfare and production challenge (245). Both prophylactic (pre-exposure) and metaphylactic (continuous exposure) delivery strategies significantly improved larval survival during *Vibrio campbellii* challenge, with dose-dependent effects. Notably, optimal protective doses differed between prophylactic and metaphylactic applications, highlighting the importance of dosage and timing for practical implementation. Protection was also observed against *V. parahaemolyticus*, the causative agent of acute hepatopancreatic necrosis disease, further supporting the broad protective potential of phloroglucinol (103). Collectively, these studies position phloroglucinol as a promising natural Hsp70 inducer with applications in disease prevention, stress mitigation, and welfare-oriented health management in aquaculture. Its capacity to enhance both immediate and long-term resilience highlights its potential value for the sustainable production of freshwater prawns and other cultured aquatic animals.

#### 8.1.3 Carvacrol

Carvacrol [2-methyl-5-[1-methylethyl]-phenol] is a major bioactive compound of oregano and thyme essential oils, and is generally recognised as a safe compound [GRAS] for use as a feed additive and food preservative in the food industry (246–248) due to its biostatic and/or biocidal effects against many bacteria, yeast, and fungi (247). Early studies in prokaryotic systems linked carvacrol exposure to increased expression of Hsp60 but not Hsp70 (248), whereas *in vitro* experiments using mammalian cells suggested that carvacrol functions as a co-inducer of Hsp70 (249). However, *in vitro* models often fail to fully reflect *in vivo* physiological responses (250). The findings obtained from *in vitro* studies, therefore, may not provide sufficient or realistic information to precisely understand the biological property [e.g., Hsp72 induction] of bioactive compounds such as carvacrol (250). To address this gap, Baruah et al. (224) examined the effects of carvacrol under *in vivo* conditions using germ-free *Artemia* as a model organism. Pretreatment with carvacrol (66.4 µM) induced a robust Hsp70 response within *Artemia*. This Hsp70-inducing effect of the compound was recorded under controlled germ-free experimental conditions, in the absence of additional stressors, demonstrating that carvacrol acts as a direct inducer of Hsp70, rather than as a co-inducer as previously reported (249). This induction was associated with enhanced resistance to subsequent thermal stress and *Vibrio harveyi* infection. Mechanistic analysis indicated that transient hydrogen peroxide generation mediates this response, identifying carvacrol as a natural pro-oxidant capable of activating Hsp70 pathways (207, 248). Despite extensive research on the nutritional, immunological, and antimicrobial effects of carvacrol in aquaculture species (251, 252), its capacity to modulate Hsp70 responses in farmed fish and shrimp has not yet been investigated. Whether Hsp70 induction contributes to the reported health benefits of carvacrol remains an important question for future research, particularly in the context of resilience-based health and welfare management in aquaculture.

#### 8.1.4 Curcumin

Curcumin is a phenolic compound naturally present in the rhizomes of the turmeric plant *Curcuma longa* and is used worldwide as a common food additive for human consumption (253). Extensive *in vitro* and *in vivo* studies have demonstrated that curcumin exhibits diverse bioactivities, including antioxidant, anti-inflammatory, antimicrobial, and immunomodulatory effects (254–262). Increasing evidence suggests that many of these beneficial effects are mediated, at least in part, through interplay with cellular stress-response pathways, particularly Hsps. Mechanistic studies have shown that curcumin can activate heat shock factor 1 (HSF1), leading to enhanced expression of Hsp70, which has been linked to its anti-inflammatory and cytoprotective properties (263–265). In aquaculture species, there is good evidence suggesting that curcumin improves the growth performances, immune status, and tolerance of aquaculture animals against abiotic and biotic stressors (266–269). Although the direct contribution of Hsp70 induction to these effects has not always been explicitly addressed, some studies provide supportive evidence. For example, dietary curcumin increased thermal tolerance in cyprinid fish, an effect attributed to the coordinated activation of Nrf2 signalling, upregulation of Hsp70 and other Hsps, and enhanced antioxidant enzyme activity (209, 264). Similarly, curcumin-fed Nile tilapia at 100–200 mg kg^-1^ of feed for 8 weeks exhibited increased total antioxidant capacity and increased hepatic expression of *hsp70* isoforms, indicating improved cellular stress resilience (248). Collectively, these findings suggest that curcumin functions as a natural Hsp-inducing compound with potential applications in aquaculture feeds to enhance stress tolerance, disease resistance, and overall animal welfare.

#### 8.1.5 Cactus extract

Extract derived from the prickly pear cactus, *Opuntia ficus indicia*, has been identified as an effective, non-invasive modulator of endogenous Hsps in animal tissues. This extract causes a state of pre-stress conditioning that primes the cellular stress response without any apparent hazardous effect, enabling a rapid and amplified induction of Hsp70 upon subsequent exposure to stress. As a result, tissue Hsp70 levels following stress are typically higher and achieved more rapidly than those induced by the stressor alone (195, 270).

Originally developed to alleviate physical fatigue in commercial divers working in seaweed farming (271), the cactus-based formulation was subsequently tested in various farmed species, including aquaculture. Experimental and field studies in poultry, rabbits, fish, and crustaceans have consistently reported beneficial effects, particularly in enhancing tolerance to biotic and abiotic stressors (**Table 1**). In aquaculture models, pretreatment with cactus extract led to rapid induction of Hsp70 in the test organisms following exposure to infection or environmental stress, with HSP levels exceeding those observed after conventional heat shock treatments (201, 272, 273).

Early studies in angelfish (*Pterophyllum scalare*) demonstrated that prior exposure of the fish to the cactus extract via immersion significantly increased Hsp70 and Hsp90 levels in liver, muscle, and gill tissues, conferring protection against otherwise lethal concentrations of toxic compounds (200). Similar protective effects were reported in common carp (*Cyprinus carpio*), where short-term pretreatment with the cactus extract improved survival under ammonia stress, coinciding with elevated Hsp70 expression in gill and muscle tissues (272). In salmon and gilthead seabream (*Sparus aurata*), pretreatment with cactus extract before experimental challenge with *Vibrio anguillarum* substantially reduced mortality compared to untreated controls (273). Together, these findings suggest that cactus-derived formulations can promote stress resilience and immune preparedness, supporting their potential inclusion in preventive health and welfare management strategies for farmed aquatic animals.

#### 8.1.6 Pandanus tectorius extract

*Pandanus tectorius* and related species are mangrove plants widely distributed in the tropical and subtropical regions. Various parts of the plant, including the fruit and leaves, are rich in bioactive compounds and have long been used as nutraceuticals in humans and animals due to their antioxidant, anticoagulant, antibacterial, and antidiabetic properties (184, 258–263). In aquaculture, crude extracts of *Pandanus* have been investigated as natural growth promoters, immunostimulants, antimicrobials, and antioxidants in several species, including *Cyprinus carpio*, *Oncorhynchus mykiss*, and *Penaeus vannamei* (184, 215, 216, 274, 275). Dietary supplementation with *Pandanus* extract has been shown to enhance immune and stress-related responses in farmed aquatic animals. For example, in *O. mykiss*, dietary administration of *Pandanus* extract at 2% inclusion level significantly upregulates the expression of immune-related genes, such as *TNF*, *LYZ2*, *IL-8*, *CD-4*, and tumour suppressor gene *WT-1a* in the head-kidney, and increased resistance towards *Yersinia ruckeri* bacterial challenge (275). Similar increment in the expression level of the genes associated with the antioxidant defence response [*SOD*, *CAT]* and inflammation [nuclear factor erythroid 2-related factor 2 and *IL-10*] has been reported in *C. carpio* due to *Pandanus* extract exposure. In penaeid shrimp as well as brine shrimp, both fruit and leaf extracts of *Pandanus* induced the expression of Hsp70 and key innate immune effectors, including prophenoloxidase, peroxinectin, penaeidin, crustin, and transglutaminase, which was associated with enhanced post-larval survival and increased resistance to *Vibrio parahaemolyticus* (184, 215, 217).

### 8.2 Inducers of heat shock response from microbial sources

#### 8.2.1 β-glucan

β-glucan is one of the most widely used immunostimulants in aquaculture, renowned for its ability to boost disease resistance in farmed aquatic species. These conserved polysaccharides, primarily derived from fungal and yeast cell walls, exert their effects mainly by modulating innate immune responses through interaction with specific pattern recognition receptors expressed on immune cells, such as macrophages, neutrophils, and natural killer cells (276–279). Besides their established immune-stimulatory effects, growing findings suggest that β-glucans can also activate the heat shock response in aquaculture species (280–282). For example, in *O. niloticus*, feeding a diet supplemented with 0.1% β-glucan led to the induction of Hsp70. Interestingly, this resulted in conferring significant protection to the fish against *Streptococcus iniae* challenge. Similar findings have been reported in giant freshwater prawn (*Macrobrachium rosenbergii*), where diets supplemented with *Saccharomyces cerevisiae* and β-glucan enhanced the expression of immune-related genes, such as prophenoloxidase, Toll-like receptors, lectins, and *hsp70* (282). In Siberian sturgeon *Acipenser baerii*, prolonged feeding with a commercial β-glucan product (MacroGard^®^) led to significantly increased *hsp70* and *hsp90* expression under sub-lethal thermal stress. In contrast, unsupplemented control groups showed a decline in *hsp70* expression (280). Considering the positive correlation between Hsp70 levels and immune responses, it appears that part of the protective effect of β-glucans in aquaculture animals may stem from their ability to induce Hsps. Consequently, β-glucans may be viewed not merely as immunostimulants but also as indirect inducers of Hsps, thereby contributing to improved stress resilience, health, and welfare in farmed aquatic species under farming conditions.

#### 8.2.2 Short-chain fatty acids

Short-chain fatty acids (SCFAs), such as acetate, propionate, butyrate, and related metabolites, are produced through anaerobic fermentation of non-digestible carbohydrates by gut microbes (283). Beyond their well-documented antimicrobial properties, SCFAs are increasingly recognised for their capacity to modulate host physiology, particularly gut health, immune function, and stress resilience (284–286). The bacteriostatic activities of SCFAs have been studied most intensively in enterobacteria such as *Salmonella* spp., *E. coli*, and *Shigella flexneri* (285). In aquaculture species, luminescent *Vibrio* spp. Showed susceptibility to SCFAs similar to enteric bacteria (286). *In vivo* challenge tests demonstrated that SCFAs significantly improved the survival of *Artemia* larvae when challenged with pathogenic *V. campbellii*, highlighting the relevance of SCFAs as functional feed components (279, 284). Importantly, SCFAs not only impacts microbes but also influence the host defense mechanisms through host-microbiota interactions (279, 280, 287, 288). An overview of the pathogen responses to SCFAs is beyond the scope of this review, as other reviews addressed this topic [for details, see review (281, 289); and (282, 290). Here, the focus is on the host responses to feeding SCFAs, with particular focus on stress proteins. Numerous studies in both terrestrial and aquaculture animals highlight the benefits of using SCFAs, especially with regard to growth promotion, better gut health and integrity, improved immunity, and increased resistance to diseases (27, 283–286, 291–294). Although the mechanisms underlying the health benefits of SCFAs in organisms are not fully understood, accumulating evidence suggests that induction of Hsps plays a central role. For instance, Ren et al. (295) reported that SCFA butyrate significantly improved the ability of rat intestinal epithelial cells to withstand oxidant injury, mediated in part by cell-specific induction of Hsp25. Similarly, in common carp, a diet enriched with microencapsulated sodium butyrate led to a notable improvement in the fish’s immunological response, which was linked to the activation of Hsp70 and both pro- and anti-inflammatory cytokines in the gut (292). In another report, the bacterial storage compound poly-ß-hydroxybutyrate [PHB], a polymer of the SCFA ß-hydroxybutyrate, was found to protect *Artemia* larvae from the virulent *V. campbellii* strain (296). Subsequent mechanistic studies demonstrated that PHB-mediated protection in *Artemia* is associated with the induction of Hsp70 and the regulation of key innate immune genes, including components of the prophenoloxidase system (142). Collectively, this evidence highlights the potential of SCFAs and related metabolites as nutritional tools for promoting health, welfare, and resilience in aquaculture through targeted modulation of Hsp responses.

## 9 Significance for aquaculture

Advances made in the field of molecular biology, immunology, and functional genomics have substantially improved our understanding of Hsp gene regulation and the downstream effects of Hsp70 on cellular protection and immune function. Over the past two decades, parallel progress in aquaculture research has demonstrated that modulation of Hsp70 expression can enhance tolerance to thermal stress, hypoxia, poor water quality, and infectious diseases in a wide range of farmed aquatic species. These findings collectively support the concept that targeting the heat shock response can shift health management strategies from reactive disease treatment toward proactive resilience building.

From a health and welfare management perspective, Hsp70-based approaches offer several advantages. By enhancing the intrinsic capacity of animals to cope with stress, Hsp70 induction may reduce disease susceptibility, limit stress-induced immune suppression, and improve performance traits such as growth, survival, and feed utilisation. Importantly, such strategies align with the growing need to reduce reliance on antimicrobials and other chemotherapeutics, while addressing the compounded pressures of farming intensification and climate-driven environmental variability. Taken together, current evidence suggests that Hsp70s and their inducers have strong potential to form part of next-generation, welfare-oriented health management tools in aquaculture.

## 10 Conclusion and future perspectives

The extensive body of evidence from experimental trials across diverse biological organisms establishes Hsps, particularly members of the Hsp70 family, and their inducers as powerful modulators of stress resilience and disease tolerance in aquaculture animals. By enhancing cellular protection, sustaining immune function, and enhancing coping mechanisms against biotic and abiotic stressors, Hsp70-based strategies offer a paradigm shift from reactive disease treatment toward preventive, welfare-oriented health management in aquaculture systems. However, translating this potential into routine aquaculture practice requires several critical challenges. Foremost among these is the need for comprehensive safety evaluations, as many Hsp inducers and co-inducers are bioactive compounds with diverse biological effects that extend beyond Hsp induction. Their impact on animal physiology, product quality, environmental integrity, and consumer health must be thoroughly assessed. Economic feasibility and practical delivery routes, particularly through functional feeds, should be carefully optimised to ensure that Hsp modulation can be implemented effectively at a commercial scale.

Another important consideration is the adaptive capacity of aquaculture pathogens towards these compounds. Although Hsp inducers primarily act by eliciting the host defence system rather than directly targeting pathogens, long-term use may still exert selective pressures that influence pathogen virulence or host–pathogen interactions. Continuous monitoring and integration with broader disease management strategies will be essential to mitigate such risks. Furthermore, a deeper understanding of the molecular pathways governing the induction of Hsp70 and other (small) Hsps, their regulation, and tissue-specific expression in aquaculture animals is needed. Such knowledge will enable the development of more targeted and efficient Hsp-inducing strategies, minimising unintended physiological trade-offs. Equally critical is the need to evaluate the long-term consequences of repeated or sustained Hsp70 induction. Future studies should investigate how chronic modulation of the heat shock response affects growth, reproduction, metabolic performance, immune balance, and overall welfare, particularly under conditions of intensified farming and climate-driven environmental variability. These insights are vital for ensuring the responsible and sustainable use of Hsp-based interventions.

Finally, the greatest benefits of Hsp70 modulation are likely to be realized when integrated with complementary management practices, including optimized nutrition, improved husbandry, biosecurity measures, and selective breeding for robustness. By embedding Hsp70-based strategies within a holistic resilience framework, the aquaculture industry can enhance animal welfare, reduce disease susceptibility, and improve production stability. Harnessing the stress-adaptive potential of Hsp70, therefore, represents a promising pathway toward more resilient, ethical, and sustainable aquaculture in the face of ongoing environmental and production challenges.
